# Effect of AKD Content on Wettability, Water Absorption Kinetics, and Interfacial Properties of Poplar Wood Flour/PLA Biocomposites

**DOI:** 10.3390/polym18212763

**Published:** 2026-11-06

**Authors:** Jiahui Zhang, Xiao Wu, Shengze Gong, Xia Yang, Hongbo Li, Zebing Xing, Yu Xian

**Affiliations:** 1 The College of Forestry, Shanxi Agricultural University, Jinzhong 030801, China; 2 The College of Agricultural Engineering, Shanxi Agricultural University, Jinzhong 030801, China

**Keywords:** alkyl ketene dimer, poplar wood flour/PLA composites, wettability, water absorption, interfacial properties

## Abstract

**Highlights:**

1. AKD treatment improved wettability and reduced surface free energy.
2. 2% AKD achieved the highest flexural strength.
3. Interfacial bonding was the key factor determining mechanical retention.

**Abstract:**

In this study, poplar wood flour was modified with alkyl ketene dimer (AKD), and poplar wood flour/polylactic acid (PLA) composites with 0–5% AKD were prepared. Effects of AKD content on wettability, water absorption kinetics, dimensional stability, flexural properties, and microscopic interface were investigated. AKD likely consumed hydroxyl groups through esterification and/or hydrogen bonding, as reported in the literature, and introduced hydrophobic alkyl chains. The 5% AKD sample exhibited the highest contact angle, while the 3% AKD sample showed the lowest total surface free energy (24.73 mJ·m^−2^). AKD content significantly changed the water diffusion mode: at 0–3% AKD, water absorption followed non-Fickian diffusion, with 2% AKD showing the highest *n* value (0.7132), indicating swelling-controlled diffusion and the lowest apparent saturation water absorption. At 4% AKD, n decreased to 0.4786, approaching Fickian diffusion, and agglomerated pores accelerated water transport. The 2% AKD sample exhibited optimal interfacial bonding and the highest flexural strength retention (46.29%). The 5% AKD sample showed the lowest steady-state thickness swelling coefficient (10.03%), indicating the best dimensional stability, but agglomeration defects weakened mechanical properties. Interfacial bonding strength, rather than low water absorption or low swelling, was key to mechanical retention. An optimal AKD content range exists when “optimal” is defined by the balance between mechanical retention and dimensional stability: 2% AKD is preferred for mechanical retention, while 5% AKD is preferred for dimensional stability under humid service scenarios. These findings support the water-resistant modification of PLA-based wood–plastic composites.

## 1. Introduction

Driven by the demand for sustainable materials and the mitigation of plastic pollution, traditional petroleum-based plastics face significant challenges due to high resource consumption and poor degradability [1,2,3]. Poplar wood flour/polylactic acid (PLA) PLA-based composites containing bio-based components combine renewability, potential biodegradability, and good mechanical properties, showing considerable application potential in construction decoration and other non-food-contact fields [4].

However, natural poplar wood flour is rich in hydrophilic hydroxyl groups and highly polar, which is poorly compatible with the weakly polar PLA matrix. This leads to weak interfacial compatibility and insufficient interfacial bonding, resulting in high water absorption, poor dimensional stability, and mechanical degradation. Therefore, interfacial modification to optimize the two-phase bonding state is key to breaking through the performance bottleneck [5,6,7].

To address this issue, researchers have established physical, chemical, biological, and synergistic modification systems. Physical modification is environmentally friendly but limited in effectiveness [8]; chemical modification is effective but consumes reagents and has environmental concerns; biological modification is mild and pollution-free but time-consuming with limited improvement [9]; synergistic modification is comprehensive but complex and costly [10]. Recent studies on lignocellulosic–filler-reinforced composites have further emphasized the balance among mechanical performance, water resistance, and filler distribution [11,12]. For example, Karagöz et al. reported that incorporating walnut shell and nanoclay into HDPE improved flexural strength from 26 to 47.9 MPa, highlighting the roles of the filler–matrix interface and filler distribution [11]. Another study on hazelnut-shell-based HDPE hybrid biocomposites demonstrated that formulation–property relationships, including water absorption below 0.36% after 240 h, are critical for evaluating structural integrity [12]. Notably, existing studies on AKD-modified wood flour/PLA composites mainly focus on mechanical properties and interfacial morphology, while systematic studies on how AKD content regulates wettability, water diffusion modes, and wet mechanical stability are still insufficient. The quantitative relationship between AKD content and water absorption kinetics parameters (n, D) and interfacial damage remains unclear. In particular, systematically varying the AKD content from 0% to 5% and quantitatively correlating it with wettability, water diffusion modes (n, D), dimensional stability, and mechanical retention represents a novel contribution of this work.

AKD is a mature green esterification sizing agent in the paper industry, with advantages such as non-toxicity, low cost, uniform modification, and no fiber damage. It has been reported to react with hydroxyl groups on plant fiber surfaces through esterification and/or hydrogen bonding, effectively shielding hydrophilic groups and reducing fiber surface polarity, thereby improving the polarity matching between wood flour and PLA matrix [13,14]. In this study, we hypothesized that an optimal AKD content range exists for balancing interfacial hydrophobicity and mechanical retention. Herein, “optimal” is defined as the AKD content that best balances mechanical retention and dimensional stability for humid service scenarios. To test this hypothesis, poplar wood flour/PLA composites were prepared at a fixed PLA-to-wood-flour mass ratio of 1:1, with AKD contents of 0%, 1%, 2%, 3%, 4%, and 5% relative to the dry wood flour mass. The wettability was characterized by dynamic contact angle and surface free energy; water absorption kinetics were analyzed using Fickian diffusion models to obtain the diffusion exponent n and apparent diffusion coefficient D; dimensional stability was evaluated by the thickness swelling coefficient; mechanical retention was assessed by flexural strength before and after water absorption; and the interfacial morphology was examined by SEM. The quantitative relationships between AKD content and these properties are reported and discussed, providing a theoretical basis and data support for the performance optimization and engineering application of AKD-modified poplar wood flour/PLA composites.

## 2. Materials and Methods

### 2.1. Materials and Equipment

Polylactic acid (PLA): 4032D: the melt flow index of PLA is approximately 10 g/10 min (190 °C, 2.16 kg) with a density of 1.25 g/cm^3^, as reported in the supplier’s technical data sheet, Dongguan Huachuang Plastic Products Co., Ltd., Dongguan, China. Poplar wood flour: 100 mesh: the chemical composition of the poplar wood flour was not separately determined in this study; poplar wood typically contains approximately 40–45% cellulose, 20–25% hemicellulose, and 20–25% lignin, as reported in the literature [15], Linyi Feixian Weichao Wood Processing Factory, Linyi, China. Alkyl ketene dimer (AKD): CAS 144245-85-2: according to the supplier, the AKD is a pale yellow waxy solid with a purity of 88%, and a melting point of >47 °C, Anhui Kuer Bioengineering Co., Ltd., Hefei, China.

Digital constant-temperature water bath: HH-2, Changzhou Longhe Instrument Manufacturing Co., Ltd., Changzhou, China; digital vernier caliper: DL91150, Xiamen Yongdaxin Electromechanical Equipment Co., Ltd., Xiamen, China; scanning electron microscope (SEM): ZEISS Sigma 300EM-30, Carl Zeiss Microscopy GmbH, Jena, Germany. Contact angle goniometer: OCA20, DataPhysics Instruments GmbH, Filderstadt, Germany; universal testing machine: STD500, Xiamen Yishite Instrument Co., Ltd., Xiamen, China; electrothermal blowing dry oven: DHG-9140A, Shanghai Yiheng Scientific Instrument Co., Ltd., Shanghai, China; high-speed mixer: FZ102, Beijing Yongguangming Medical Instrument Co., Ltd., Beijing, China; extruder: CV22, Yichuan Gear Motor Co., Ltd., Luoyang, China; flat plate vulcanizing press: ST-15YP, Kunshan Lugong Precision Instrument Co., Ltd., Kunshan, China.

### 2.2. Composite Preparation

Poplar wood flour and PLA were dried in a blowing dry oven at 80 °C for 12 h. The materials were mixed uniformly in a high-speed mixer at a PLA-to-poplar wood flour mass ratio of 1:1.

Preparation of AKD-modified poplar wood flour: AKD was weighed at 0%, 1%, 2%, 3%, 4%, and 5% of the poplar wood flour mass, dissolved in a small amount of anhydrous ethanol, and uniformly sprayed onto the surface of the dried poplar wood flour. The mixture was then dried at 80 °C to constant weight to obtain AKD-modified poplar wood flour.

The modified poplar wood flour and PLA were mixed uniformly at a mass ratio of 1:1 and then extruded and pelletized using a twin-screw extruder. The extrusion process parameters were as follows: feeding temperature, 50 °C; barrel temperature, 155 °C; die temperature, 145 °C.

After pelletizing, compression molding was performed at a molding temperature of 170 °C, a molding pressure of 4 MPa, and five holding cycles, each lasting 6 min.

### 2.3. Performance Testing

(1) Contact Angle and Surface Free Energy Measurements

Contact angles were measured at room temperature (25 ± 1 °C). The droplet volume was 3 μL, and the camera-to-sample distance was fixed at approximately 10 cm. For each test liquid, at least five different positions on each sample surface were measured (one droplet per position), and the results are reported as mean ± standard deviation. The dynamic contact angle of the composites was recorded from 0 to 300 s. Contact angle–time curves were plotted for three test liquids: formamide, glycerol, and distilled water. The 300 s apparent steady-state contact angle was taken and imported into SCA20 software. The surface free energy and its dispersive and polar components were calculated based on the Owens–Wendt–Rabel–Kaelble (OWRK) model. The OWRK model equations are as follows:

(1) $$\gamma_{L}(1+\cos\theta )=2\sqrt{\gamma_{S}^{d}\gamma_{L}^{d}}+2\sqrt{\gamma_{S}^{p}\gamma_{L}^{p}}$$

(2) $$\gamma_{S}=\gamma_{S}^{d}+\gamma_{S}^{p}$$

where γ_L_ is the total surface tension of the test liquid (mJ·m^−2^); θ is the apparent steady-state contact angle of the liquid on the specimen surface (°); $\gamma_{S}^{d}$ is the dispersive component of the solid surface free energy (mJ·m^−2^); $\gamma_{S}^{p}$ is the polar component of the solid surface free energy (mJ·m^−2^); $\gamma_{L}^{d}$ is the dispersive component of the test liquid surface tension (mJ·m^−2^); and $\gamma_{L}^{p}$ is the polar component of the test liquid surface tension (mJ·m^−2^).

The γ_L_, $\gamma_{L}^{d}$, and $\gamma_{L}^{p}$ values of formamide, glycerol, and distilled water are known constants (Table 1). The 300 s steady-state average contact angles measured with the three liquids were substituted into the equations, and the dispersive component $\gamma_{S}^{d}$, polar component $\gamma_{S}^{p}$, and total solid surface free energy γ_S_ were obtained by linear fitting.

(2) Water Absorption and Dimensional Stability Tests

Water absorption tests were conducted according to ASTM D570-98. The specimen dimensions were 76.2 mm × 25.4 mm × 2.2 mm. At room temperature, the specimens were dried to constant weight and then fully immersed in deionized water. They were removed at predetermined time intervals, and surface water was wiped off. The mass and thickness were measured at different immersion times, and the instantaneous water absorption and thickness swelling coefficient were calculated. The 216 h immersion result was used to characterize the saturated water absorption state, and the variation in water absorption performance with immersion time was investigated. The instantaneous water absorption m_t_ at time t was calculated as follows:

(3) $$m_{t}=\frac{G_{t}-G_{0}}{G_{0}}\times 100\%$$

where m_t_ is the instantaneous water absorption at time t (%); G_0_ is the initial dry mass of the specimen (g); and G_t_ is the wet mass of the specimen after immersion for t hours (g).

The instantaneous thickness swelling coefficient β_t_ and the 216 h steady-state thickness swelling coefficient β_∞_ were calculated as follows:

(4) $$\beta_{t}=\frac{H_{t}-H_{0}}{H_{0}}\times 100\%$$

(5) $$\beta_{\infty }=\frac{H_{216}-H_{0}}{H_{0}}\times 100\%$$

where H_0_ is the initial thickness of the dry specimen (mm); H_t_ is the measured thickness of the specimen after immersion for t hours (mm); H_216_ is the steady-state thickness after 216 h immersion (mm); β_t_ is the instantaneous thickness swelling coefficient (%); and β_∞_ is the steady-state thickness swelling coefficient (%).

(3) Flexural Strength Test

Flexural strength was tested according to ASTM D790-10 at 0, 12, 24, 72, 120, 168, 216, 264, 336, and 480 h. The specimen dimensions were 80 mm × 12.7 mm × 2.2 mm, the support span was 80 mm, and the test speed was 2 mm/min. Five specimens were tested for each group, and the average value was taken.

(4) Scanning electron microscope (SEM)

The interfacial morphology of dry unimmersed specimens and flexural fracture surfaces after 216 h of saturated water absorption were observed using a scanning electron microscope (SEM). The samples were sputter-coated with gold, and the accelerating voltage was 5 kV. The interfacial micromorphology between the wood flour and PLA matrix was observed, and images were collected.

## 3. Results and Discussion

### 3.1. Effect of AKD on Wettability

**Figure 1:**
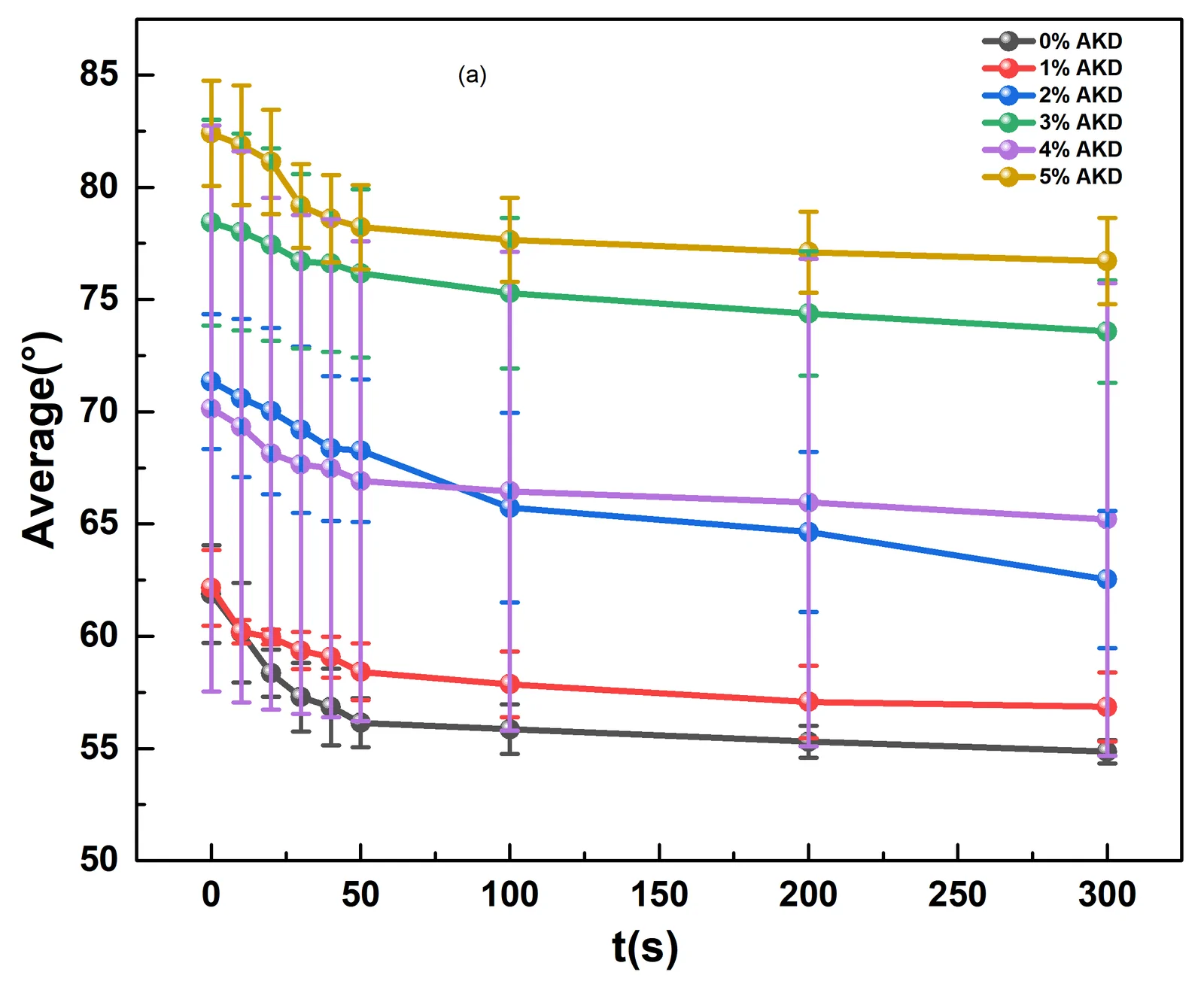

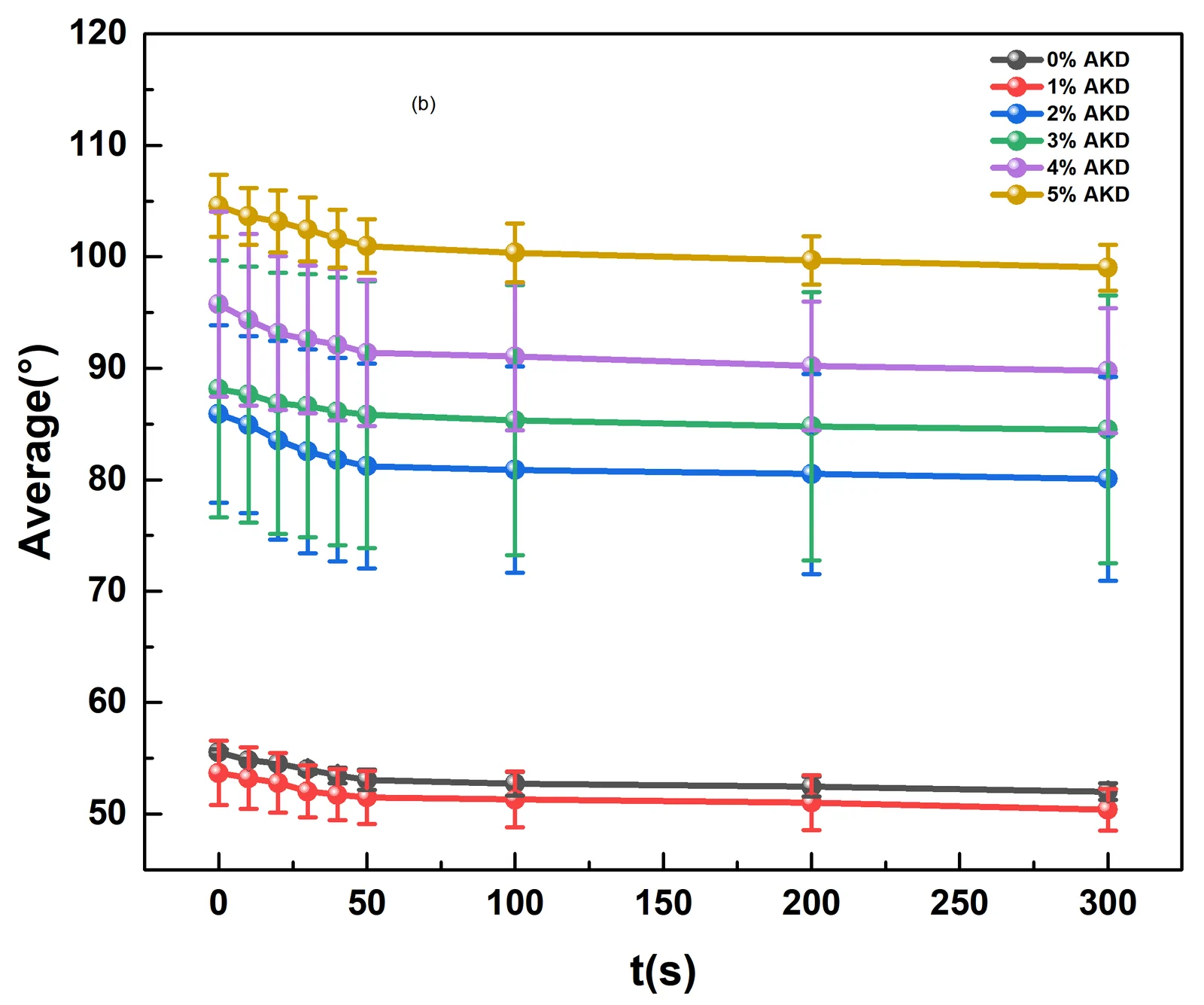

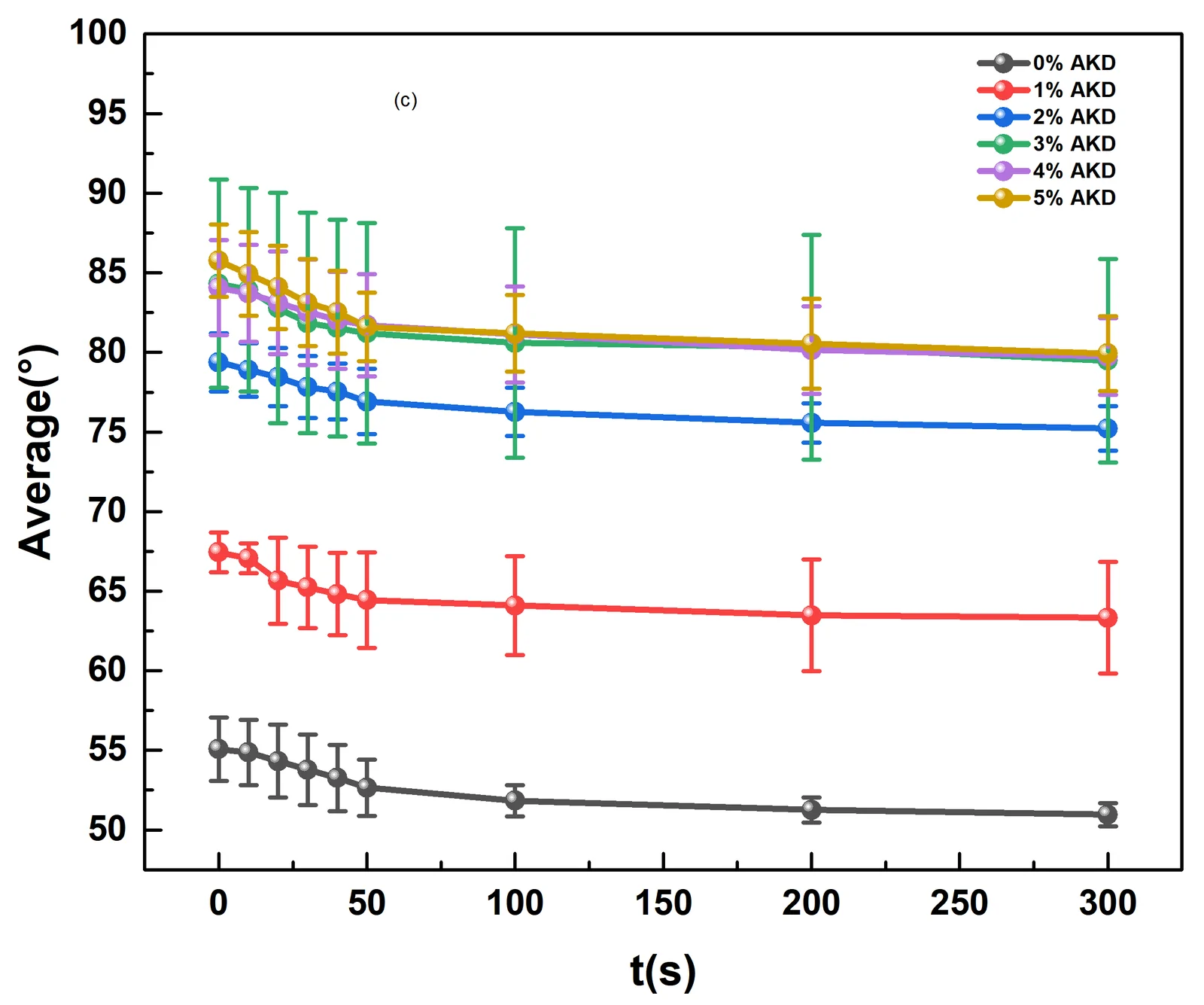
Dynamic contact angle curves of formamide, glycerol, and distilled water on the surfaces of composites with different AKD contents. (**a**) formamide, (**b**) glycerol, and (**c**) distilled water.

Figure 1 shows the dynamic contact angle–time curves of the composites with different AKD contents on the surface using three different test liquids: formamide, glycerol, and distilled water. Here, (a) is formamide, (b) is glycerol, and (c) is distilled water.

Under the same test liquid, the 300 s apparent steady-state contact angle generally increased with increasing AKD content, but differences existed among liquids at low AKD contents. The lowest apparent steady-state contact angles for formamide and glycerol occurred at 1% AKD, with values of 56.01° and 51.59°, respectively, while the lowest value for distilled water occurred at the unmodified sample at 48.35°. When the AKD content increased to 2–5%, long-chain alkyl groups formed a continuous low-surface-energy alkyl barrier on the material surface, and the contact angles of all three liquids increased synchronously. The 5% AKD sample exhibited the highest contact angles throughout the test period, approximately 80°, 98°, and 82°, respectively, indicating the strongest reduction in wettability. At the low AKD content of 1%, a complete hydrophobic coating could not be formed. Formamide and glycerol, with higher viscosity, readily underwent capillary penetration and local swelling along surface micropores, offsetting the limited hydrophobic effect introduced by the small amount of AKD [17,18]. Distilled water was mainly governed by hydroxyl hydrogen bonding, and the unmodified sample, with the strongest surface polarity, exhibited the lowest contact angle [19].

For all formulations, the contact angles of the three liquids showed a rapid decrease from 0 to 100 s followed by gradual stabilization from 100 to 300 s, with more pronounced decay at low AKD contents. It should be noted that the wood flour/PLA composite surfaces have certain porosity and water absorbency; therefore, the decrease in contact angle over time may include both liquid spreading and liquid absorption. The 300 s value is referred to as the “apparent steady-state” contact angle rather than a strict thermodynamic equilibrium contact angle. Under formamide, the 1% AKD sample exhibited the largest decay (9.72%); under glycerol, the 2% AKD sample showed the largest decay (6.86%); and under distilled water, the unmodified sample exhibited the largest decay (8.22%). These differences are related to the physicochemical properties of the test liquids and the swelling behavior of the AKD-modified surface layer. Formamide has high polarity and high surface tension, leading to stronger penetration and swelling effects on the surface layer of the 1% AKD sample. Glycerol has high viscosity and spreads slowly, readily causing a sustained swelling effect on the 2% AKD sample surface. Distilled water most significantly penetrated the native pores of the unmodified sample, causing obvious swelling [20]. With increasing AKD content, the curves became flatter, and the contact angle decay was significantly suppressed.

As reported in the literature, AKD likely consumed the polar hydroxyl groups on the poplar wood flour surface through esterification, and hydrophobic alkyl chains were enriched on the surface layer, effectively weakening the continuous wetting effect of formamide, glycerol, and distilled water on the material surface [21]. For the same sample, the apparent steady-state contact angle generally followed the order glycerol > water > formamide, as shown in Figure 1. This sequence cannot be explained by liquid polarity alone; it also reflects the combined effects of surface tension and viscosity. Glycerol has a relatively high viscosity and spreads slowly, which limits rapid penetration and results in a higher apparent contact angle. Distilled water has a high surface tension and a large polar component, leading to stronger wetting of the polar composite surface. Formamide has a lower surface tension but high polarity, which promotes spreading and penetration, giving the lowest contact angle. AKD modification effectively weakened the spreading behavior of polar liquids on the composite surface, achieving a reduction in surface wettability.

According to Equations (1) and (2), the surface free energy parameters of the composites are shown in Table 2.

As shown in Table 2, the unmodified sample exhibited the highest total surface free energy *γ_S_* (53.20 mJ·m^−2^), with a polar component $\gamma_{S}^{p}$ of 47.14 mJ·m^−2^ and a dispersive component $\gamma_{S}^{d}$ of only 6.06 mJ·m^−2^. This indicates that the unmodified poplar wood flour surface exposed numerous polar hydroxyl groups, and the system was dominated by polar interactions. These polar sites readily formed hydrogen bonds with polar water molecules and test liquids, corresponding to the consistently low dynamic contact angles and significant wetting phenomenon [22].

With increasing AKD content, the total surface free energy *γ_S_* generally decreased: to 38.72 mJ·m^−2^ at 1% AKD, to 27.30 mJ·m^−2^ at 2% AKD, and stabilized at 24.73–24.95 mJ·m^−2^ at 3–5% AKD. As reported in the literature, the lactone groups in AKD molecules likely reacted with the hydroxyl groups on the poplar wood flour surface through esterification and/or hydrogen bonding [21], continuously consuming or shielding polar active sites and reducing the polar component $\gamma_{S}^{p}$ from 47.14 mJ·m^−2^ for the unmodified sample to 14.47 mJ·m^−2^ at 2% AKD, which was the main contribution to the decrease in total surface free energy.

The dispersive component $\gamma_{S}^{d}$ showed a non-monotonic trend: it increased to 20.42 mJ·m^−2^ at 1% AKD, the highest among all groups; decreased to 8.90–12.96 mJ·m^−2^ at 2–4% AKD; and reached the lowest value (2.42 mJ·m^−2^) at 5% AKD. The abnormal increase in $\gamma_{S}^{d}$ at 1% AKD may be related to the discrete distribution of AKD on the wood flour surface and partial exposure of long-chain alkyl groups. At 5% AKD, $\gamma_{S}^{d}$ decreased significantly while $\gamma_{S}^{p}$ increased to 22.53 mJ·m^−2^, indicating that excessive AKD agglomeration led to an uneven low-surface-energy alkyl layer and re-exposure of some polar groups [23]. This is consistent with the aforementioned phenomenon that the 5% AKD sample had a high contact angle but complex decay behavior.

As reported in the literature, AKD modification likely consumed polar hydroxyl groups through esterification and/or hydrogen bonding and introduced hydrophobic alkyl chains, significantly reducing the total surface free energy of the composites and decreasing the relative contribution of polar interactions to surface wettability [24], which is consistent with the dynamic contact angle test results.

### 3.2. Water Absorption Behavior of AKD-Modified Composites

#### 3.2.1. Water Absorption Behavior and Diffusion Kinetics

**Figure 2:**
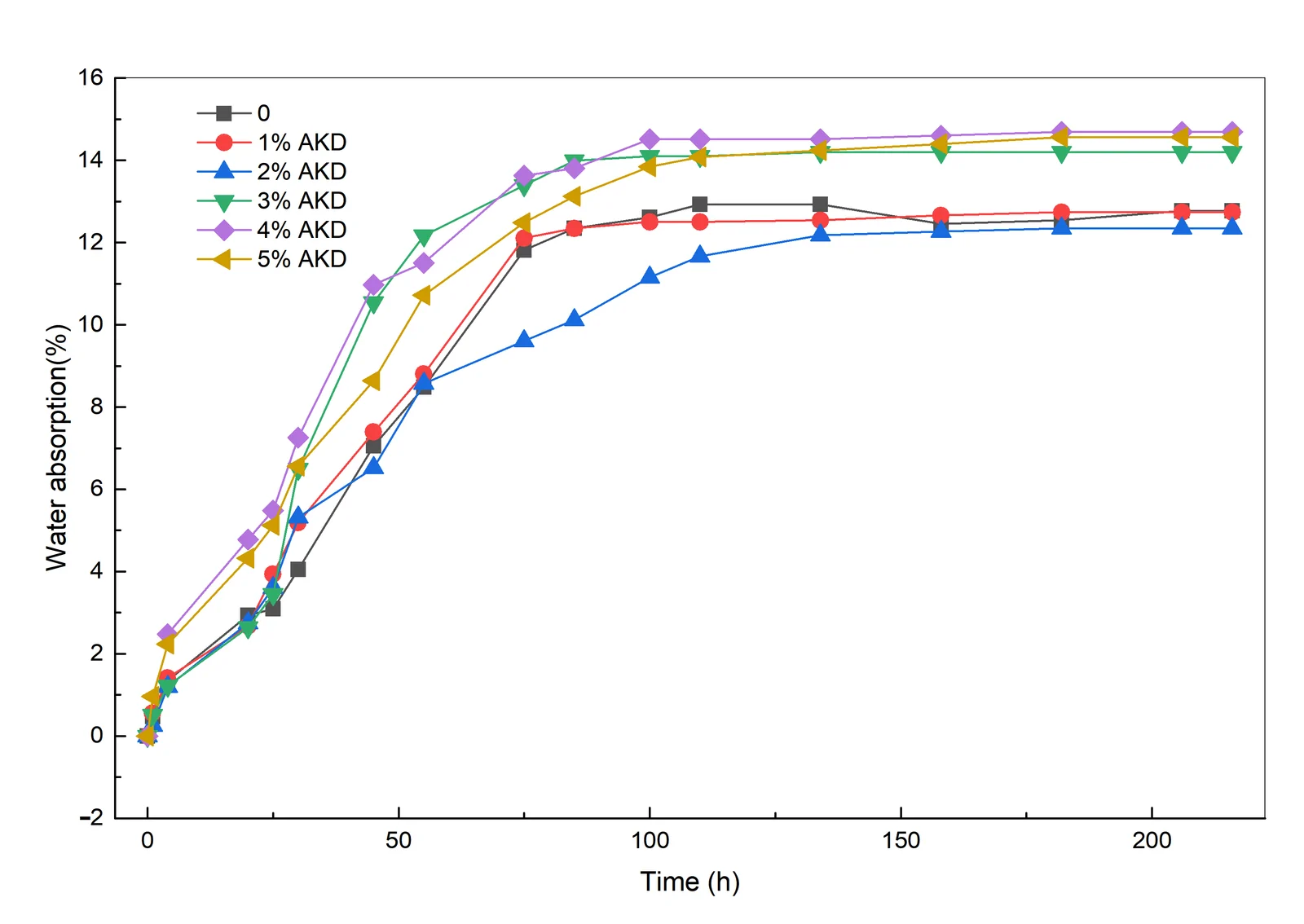
Water absorption–time curves of composites with different AKD contents.

The water absorption of poplar wood flour/PLA composites was calculated according to Equation (3), and the water absorption–time curves are shown in Figure 2. As shown in Figure 2, the water absorption curves leveled off after approximately 200 h, and the values at 264–480 h changed by less than 2% relative to 216 h; therefore, 216 h was taken as the apparent saturation point.

(1) Variation in apparent saturation water absorption with AKD content

At the same immersion time, AKD content significantly affected the water absorption of the composites. The 2% AKD sample exhibited the lowest apparent saturation water absorption, indicating the best hydrophobic modification effect. The unmodified and 1% AKD samples showed moderate water absorption. When AKD content increased to 3–5%, the water absorption continuously increased, and the 4% AKD sample reached the maximum value.

These trends are closely related to the dispersion state of AKD on the wood flour surface and the interfacial structure [25]. At low-to-medium AKD contents (1–3%), AKD likely consumed the hydroxyl groups on the wood flour surface through esterification and/or hydrogen bonding, as reported in the literature [24], and introduced hydrophobic alkyl chains, while filling the interfacial pores between wood flour and PLA, hindering water penetration. At 2% AKD, the interfacial bonding was optimal and the porosity was minimal, resulting in the lowest water absorption. When AKD content exceeded 3%, excessive AKD agglomerated on the wood flour surface, forming new interfacial defects and internal pores between the agglomerates and the PLA matrix, which provided fast transport channels for water and led to a rebound in water absorption. The 4% AKD sample exhibited the highest water absorption due to the most significant agglomeration defects. At 5% AKD, part of the AKD migrated to the surface, improving surface hydrophobicity (consistent with the increase in $\gamma_{S}^{p}$ to 22.53 mJ·m^−2^ for the 5% AKD sample in Section 2.1), but internal defects remained, resulting in slightly lower water absorption than the 4% AKD sample.

In terms of time evolution, all samples exhibited a rapid increase in water absorption from 0 to 100 h, with water rapidly penetrating into the material. After 100 h, the water absorption rate slowed down, and the curves gradually stabilized, approaching saturation. Samples with higher AKD contents showed a steeper initial slope and faster water absorption rate, which is related to the fast water channels formed by agglomeration defects [26].

(2) Early-stage water absorption kinetics and diffusion mode determination

The early-stage water absorption–time curves were fitted using the power–law model:

(6) $$\frac{m_{t}}{m_{216}}=K\cdot t^{n}$$

where *m_t_* is the water absorption at time t (%); *m*_216_ is the apparent saturation water absorption at 216 h (%); *K* is the water absorption rate constant; *n* is the diffusion characteristic exponent; and t is the immersion time in hours (h). The fitting was restricted to the early stage ($\frac{m_{t}}{m_{216}}$ ≤ 0.6).

The common logarithm of both sides of Equation (6) is taken as follows:

(7) $$\mathrm{lg}(\frac{m_{t}}{m_{216}})=\mathrm{lgK}+\mathrm{nlgt}$$

Using *lgt* as the abscissa and $\mathrm{lg}(\frac{m_{t}}{m_{216}})$ as the ordinate, the diffusion exponent n, intercept *lgK*, and goodness of fit R^2^ were obtained by least-squares linear fitting, as shown in Table 3. The criterion for ideal Fickian diffusion is n ≈ 0.5 [27]; when 0.5 < n < 1, water diffusion is coupled with polymer matrix swelling relaxation, belonging to non-Fickian anomalous diffusion; when n ≈ 1, it is Case II diffusion.

As shown in Table 3, the diffusion exponent n of the 0%, 1%, and 3% AKD samples ranged from 0.62 to 0.68, indicating non-Fickian anomalous diffusion, which suggests that water transport was jointly controlled by concentration gradient and wood flour swelling. The 2% AKD sample exhibited the highest n value (0.713), indicating the strongest non-Fickian anomalous diffusion effect, with swelling-controlled diffusion dominating the water absorption process. The 4% AKD sample had an n value of 0.479, approaching Fickian diffusion. The 5% AKD sample had an n value of 0.531, which, although still in the non-Fickian range, was close to Fickian diffusion. All samples showed R^2^ values greater than 0.90, indicating good fitting quality. Residual analysis showed no systematic trend, with residuals randomly distributed around zero (Figure S1).

(3) Initial apparent diffusion coefficient analysis

Based on the early-stage-stage water absorption data, except for the 4% AKD sample which approached Fickian diffusion, the other samples exhibited non-Fickian anomalous diffusion overall. However, in the initial stage of water absorption ($\frac{m_{t}}{m_{216}}$ ≤ 0.6), water had not yet caused significant interfacial damage or matrix relaxation, and the water absorption process approximately satisfied the Fickian short-time linear relationship [28]. To quantitatively compare the relative initial water diffusion rates of different samples, Fick’s second law short-time approximation model was used to linearly fit the initial data, and the apparent diffusion coefficient D was calculated. It should be noted that this D value reflects the apparent diffusion rate in the initial stage, rather than the strict Fickian diffusion coefficient over the entire stage.

Fick’s second law short-time approximation model is as follows:

(8) $$\frac{m_{t}}{m_{216}}=\frac{4\sqrt{D}}{\sqrt{\pi }H_{0}}\sqrt{t}$$

**Figure 3:**
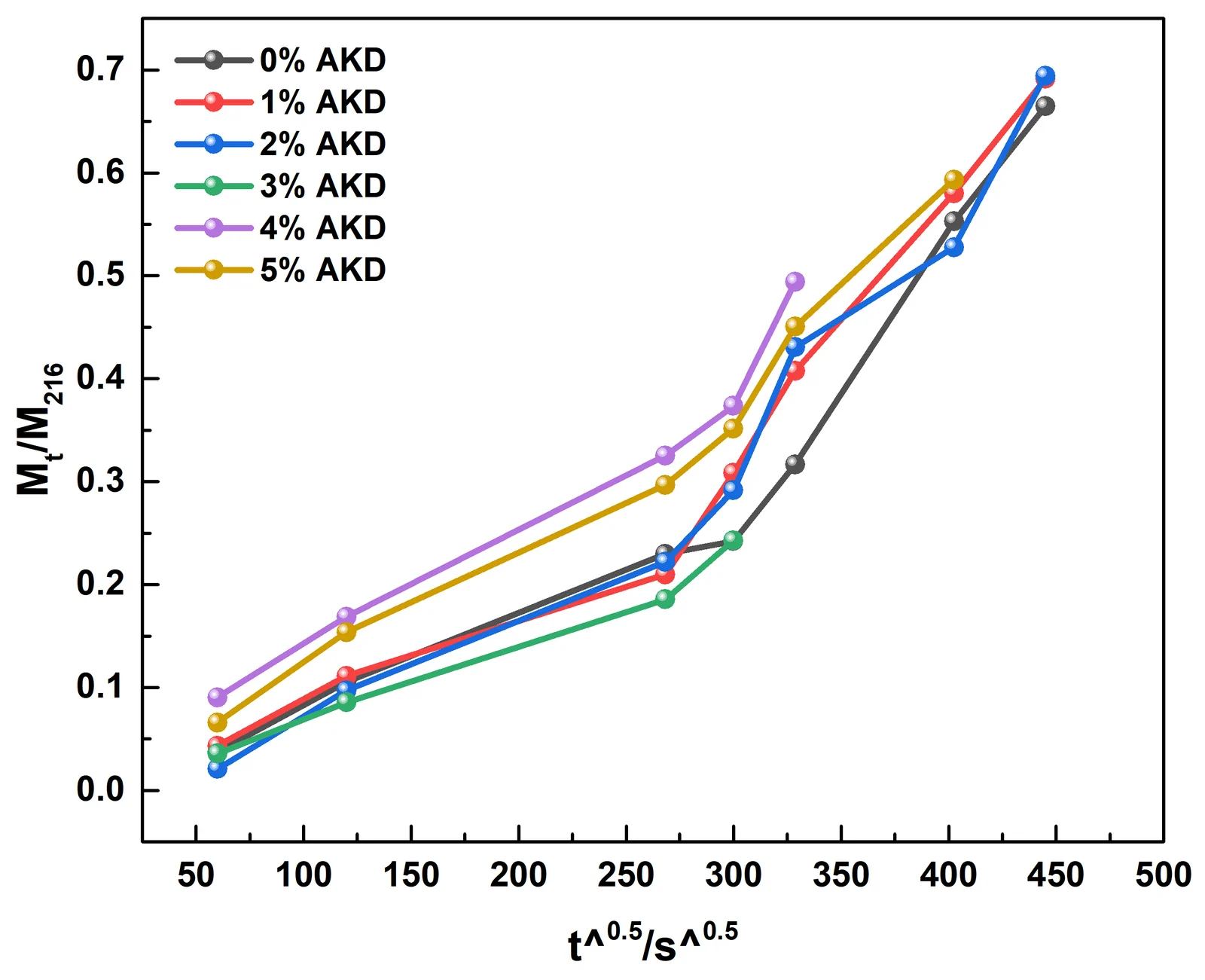
Fitting curves of relative water absorption versus square root of time for composites with different AKD contents.

Using the square root of immersion time $\sqrt{t}$ as the abscissa and the relative water absorption $\frac{m_{t}}{m_{216}}$ as the ordinate, the plot was made. To eliminate data interference from matrix swelling relaxation in the later stage, only the initial water absorption data with $\frac{m_{t}}{m_{216}}$ ≤ 0.6 were selected for linear fitting, as shown in Figure 3. The cutoff of $\frac{m_{t}}{m_{216}}$ ≤ 0.6 was chosen because the Fickian short-time approximation is generally valid only in the early stage, and 0.6 is a commonly used upper limit in wood–plastic composite studies [27]. The same cutoff was applied to all formulations to ensure comparability.

Longitudinally, in the early stage of water absorption, $\frac{m_{t}}{m_{216}}$ was approximately linear with $\sqrt{t}$, and all samples exhibited Fickian diffusion characteristics in the initial stage. The 2% AKD sample showed the largest slope, indicating the fastest initial water diffusion rate. The 3% AKD sample exhibited the smallest slope, indicating the lowest initial diffusion rate. The unmodified and 1% AKD samples were at intermediate levels. When AKD content increased to 4–5%, the slope increased, and the water diffusion rate accelerated.

According to Equation (8), the apparent diffusion coefficient *D* of each sample was calculated from the fitted slope *k*:

(9) $$D=\frac{\pi k^{2}H_{0}^{2}}{16}$$

where k is the slope obtained from the linear fitting of $\sqrt{t}$ versus $\frac{m_{t}}{m_{216}}$; H_0_ is the initial thickness of the specimen (mm); and *D* is the apparent diffusion coefficient (mm^2^·s^−1^).

The calculation results are shown in Table 4.

As shown in Table 4, the 2% AKD sample exhibited the highest initial apparent diffusion coefficient (2.76 × 10^−6^ mm^2^·s^−1^), followed by the 1% AKD sample (2.62 × 10^−6^ mm^2^·s^−1^), while the unmodified sample was also relatively high (2.49 × 10^−6^ mm^2^·s^−1^). The 3% AKD sample decreased to the lowest value (1.64 × 10^−6^ mm^2^·s^−1^). The 4% and 5% AKD samples showed intermediate values of 2.07 × 10^−6^ and 2.32 × 10^−6^ mm^2^·s^−1^, respectively.

It should be noted that the 2% AKD sample had the highest initial apparent diffusion coefficient but the lowest water absorption at 216 h (see Section 3.2.1). This seemingly contradictory phenomenon can be attributed to the two-stage diffusion characteristics of “rapid initial penetration–later swelling blockage”: in the initial stage, water rapidly caused wood flour swelling, and the swelling stress opened the interface to form new water channels [29], resulting in a relatively high initial apparent diffusion coefficient. However, as swelling continued, the pores were blocked [30], and later water advancement was hindered, so the water absorption at 216 h remained the lowest. The 3% AKD sample had the lowest initial diffusion coefficient, indicating that this AKD content optimized the interfacial structure, increased water diffusion resistance, and minimized the initial diffusion rate. The higher D values for 0% and 1% AKD are consistent with their numerous native interfacial gaps, while the intermediate D values for 4% and 5% AKD reflect the coexistence of agglomeration pores and surface hydrophobicity.

For the 0–3% AKD samples, the interfacial compatibility between the wood flour and PLA matrix fluctuated considerably, and interfacial debonding and microcracks developed randomly during water absorption, resulting in stronger dispersion of water absorption kinetics and relatively lower fitting goodness R^2^ for some samples (0.6963–0.9111). Specifically, the relatively low R^2^(0.6963) for the 3% AKD sample indicates greater scatter in the initial-stage data, so its D value should be interpreted with caution. For the 4% AKD sample, the “Near-Fickian” classification in Table 3 is based primarily on the n value (0.479) rather than on R^2^(0.8656) alone.

#### 3.2.2. Swelling Behavior Analysis

The thickness swelling coefficient β was used to evaluate the dimensional stability of the composites in the thickness direction after water absorption. A lower β value indicates better resistance to water-induced deformation and dimensional stability. The instantaneous thickness swelling coefficient β_t_ and the 216 h steady-state thickness swelling coefficient β∞ were calculated according to Equations (4) and (5).

According to Equation (4), the instantaneous thickness swelling coefficients of each sample under different immersion times are shown in Table 5.

(1) Time evolution of thickness swelling

As shown in Table 5, the thickness swelling coefficients of all samples increased rapidly with immersion time in the early stage. At 1 h, the thickness swelling coefficients of all samples were only 0.25–0.33%. The swelling reached a peak at 85 h, e.g., 13.96% for the 1% AKD sample. After 85 h, the thickness swelling coefficient decreased and then stabilized after 134 h, reaching swelling equilibrium at 216 h. The slight decrease in thickness swelling after the peak (e.g., 1% AKD: 13.96% at 85 h → 12.72% at 216 h) is attributed to stress relaxation of the PLA matrix and partial collapse/filling of the initially opened interfacial pores, with minor contributions from extractive leaching and measurement variation [14].

(2) Effect of AKD content on thickness swelling

At the same immersion time, taking 55 h as an example, the thickness swelling coefficients of the 1–3% AKD samples ranged from 10.70% to 10.91%, which were relatively high. In this content range, the two-phase interface was improved, and the wood flour swelling effect was significant after water penetration. The 5% AKD sample exhibited the lowest thickness swelling coefficient (8.98%), indicating that the hydrophobic effect of high AKD content effectively inhibited water-induced swelling of wood flour. With further extension of immersion time, the thickness swelling of all groups gradually stabilized, reaching swelling equilibrium at 216 h.

(3) Steady-state thickness swelling coefficient

As shown in Table 5, the 216 h steady-state thickness swelling coefficients β∞ of the 0–5% AKD samples were 11.61%, 12.72%, 12.37%, 12.37%, 11.30%, and 10.03%, respectively. β∞ showed a trend of first increasing and then decreasing with AKD content. The 1% AKD sample had the highest β∞, followed by the 2% and 3% AKD samples, while the 0% AKD sample was intermediate, the 4% AKD sample was lower, and the 5% AKD sample was the lowest.

The regulation of thickness swelling by AKD content involves a dual mechanism: at low-to-medium contents (1–3%), AKD improved interfacial bonding and reduced native pores, so water mainly induced wood flour swelling, resulting in larger thickness swelling. At high contents (4–5%), the hydrophobic effect of AKD increased, reducing water ingress, while agglomeration defects caused part of the water to fill pores rather than induce swelling, thus reducing thickness swelling [31,32].

#### 3.2.3. Mechanical Properties After Water Absorption

**Figure 4:**
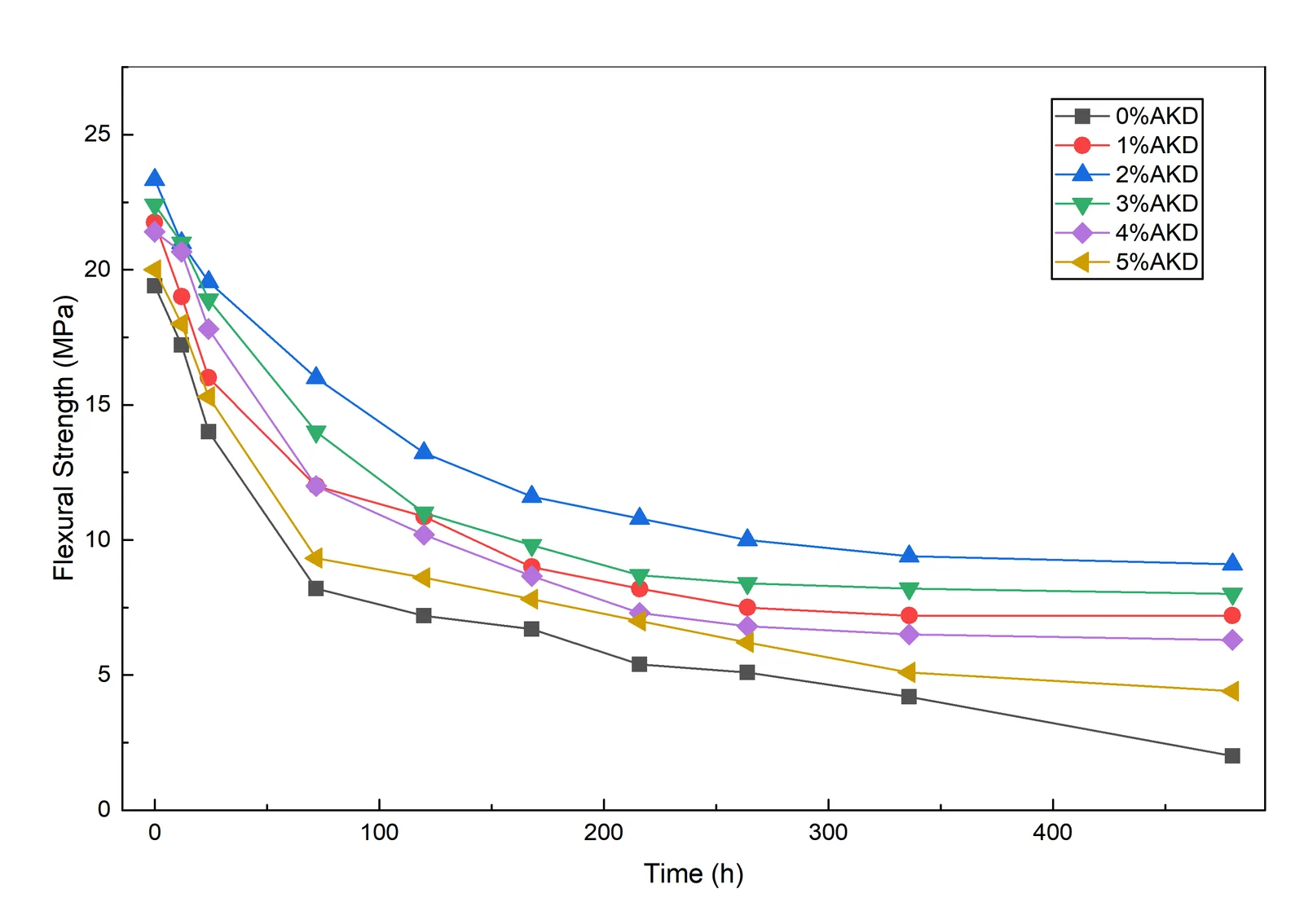
Flexural strength as a function of immersion time.

Figure 4 shows the variation in flexural strength of the composites with immersion time.

(1) Effect of AKD content on mechanical retention

Longitudinally, the 2% AKD sample exhibited the best flexural strength retention and the highest residual flexural strength after saturated water absorption. The 0% unmodified sample showed the lowest retention and the most obvious mechanical degradation. Although the 5% AKD sample possessed hydrophobic characteristics, the internal defects caused by AKD agglomeration made its mechanical retention weaker than that of the 2% AKD group. The flexural strength retention of each sample after saturated water absorption (216 h) is shown in Table 6.

As shown in Table 6, with increasing AKD content, the flexural strength retention first increased and then decreased. The 2% AKD sample exhibited the highest retention (46.29%), which was 18.45 percentage points higher than that of the unmodified sample (27.84). An appropriate AKD content optimized interfacial bonding and alleviated the mechanical degradation caused by water absorption. However, excessive AKD content led to agglomeration, and the retention decreased. The dry flexural strength of the present composites (19.40–23.33 MPa) is somewhat lower than that reported for walnut-shell/nanocellulose/HDPE systems (26–47.9 MPa) in [11]. However, the focus of this study is the flexural strength retention after water absorption rather than the absolute dry strength, because retention is more relevant to humid service conditions. For the 2% AKD sample, the wet strength was 10.80 MPa (loss of 12.53 MPa from 23.33 MPa), while the unmodified sample showed only 5.40 MPa (loss of 14.00 MPa from 19.40 MPa), so 2% AKD had both the highest absolute wet strength and the smallest absolute loss. The 2% vs. 5% difference should not be judged solely from retention ratios, since 2% AKD also had higher absolute wet strength (10.80 vs. 7.00 MPa) and smaller loss (12.53 vs. 13.00 MPa). Flexural strength was measured to 480 h, but the ranking remained unchanged after 216 h, which also matches the apparent saturation point, so 216 h was used as the common comparison point.

(2) Two stages of mechanical degradation

Laterally, the flexural strength of all samples decreased continuously with increasing immersion time, and the degradation process could be roughly divided into two stages. In the rapid degradation stage (0–100 h), water molecules rapidly diffused into the matrix, the poplar wood flour swelled, and the wood flour/PLA interface debonded. Interfacial damage accumulated rapidly, and the flexural strength decreased quickly. In the plateau degradation stage (after 100 h), water diffusion basically reached saturation; the development of interfacial damage slowed down; and the flexural strength entered a slow declining plateau, gradually stabilizing. This two-stage degradation trend is consistent with the stage characteristics of the water absorption–time curves in Section 3.2.1 (“rapid increase from 0 to 100 h, then slowing down after 100 h”), indicating that water diffusion and matrix swelling are the core factors inducing interfacial damage and mechanical deterioration.

Combined with the thickness swelling results in Section 3.2.2, the 2% AKD sample had a β∞ of 12.37%, which was not the lowest, but its flexural strength retention was the best. The 5% AKD sample had the lowest β∞ (10.03%), but its retention was lower than that of the 2% AKD sample. This indicates that the degree of thickness swelling cannot fully determine the extent of mechanical degradation, and interfacial bonding strength may be a more critical factor [33,34]. A more detailed mechanism analysis will be comprehensively discussed in Section 3.2.5 combined with the SEM interfacial morphology.

#### 3.2.4. Interfacial Morphology Before and After Water Absorption

**Figure 5:**
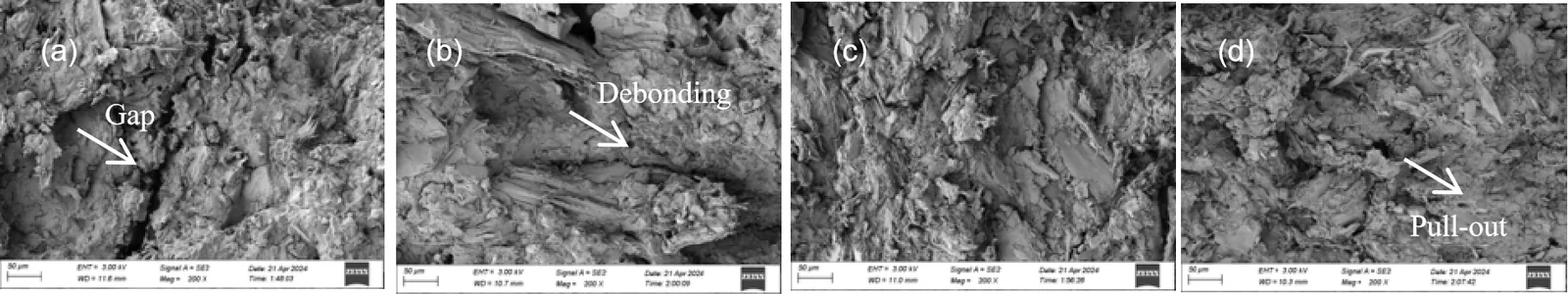
SEM images of AKD-modified poplar wood flour/PLA composites: (**a**) unmodified dry state; (**b**) unmodified after saturated water absorption; (**c**) 2% AKD dry state; (**d**) 2% AKD after saturated water absorption.

Figure 5 shows SEM images of the flexural fracture surfaces of the unmodified and 2% AKD composites before and after water absorption.

In the dry state, the unmodified sample (Figure 5a) showed clear interfacial gaps and fiber pull-out holes, indicating poor interfacial compatibility. After AKD modification (Figure 5c), the interfacial gaps were reduced and fiber pull-out was weakened, indicating improved interfacial compatibility.

After saturated water absorption, the unmodified sample (Figure 5b) showed expanded interfacial pores and severe debonding, consistent with its higher water absorption and thickness swelling. The 2% AKD sample (Figure 5d) still maintained relatively good interfacial bonding, with less hole formation and fiber pull-out, consistent with its higher flexural strength retention in Section 3.2.3. The SEM analyses of lignocellulosic–filler composites in [11,12] also emphasized the influence of filler distribution and interfacial features on performance. The interfacial gaps, fiber pull-out, and water-induced debonding observed in this study are consistent with those reports.

It should be noted that SEM characterization was performed only for the unmodified and 2% AKD samples as representative cases. The interfacial states of the other AKD contents were inferred from water absorption kinetics and mechanical retention rather than directly observed by SEM. Further SEM characterization of the full AKD range would strengthen the interfacial analysis.

#### 3.2.5. Comprehensive Analysis of Water Absorption Failure Mechanism

The water absorption kinetics parameters (n, D), 216 h steady-state thickness swelling coefficient (β_∞_), flexural strength retention after saturation, and water absorption mechanisms of each sample are summarized in Table 7.

As shown in Table 7, the regulation of water absorption failure by AKD content is jointly determined by interfacial bonding strength, surface hydrophobicity, and agglomeration defects. The water absorption kinetics parameters (n, D) are not simply linearly related to β∞ or mechanical retention.

The 2% AKD sample had the highest n (0.713) and the highest D (2.76 × 10^−6^ mm^2^·s^−1^), but its β∞ was not the lowest (12.37%), while its flexural strength retention was the highest (46.29%). This is because its interfacial bonding was superior to that of the unmodified sample (supported by Figure 5c,d); although water rapidly entered and caused swelling in the initial stage, the tight interface constrained debonding damage, and later swelling blocked the pores, resulting in the lowest apparent saturation water absorption. The 5% AKD sample had the lowest β∞ (10.03%), but its mechanical retention (35.00%) was lower than that of 2% AKD, indicating that optimal dimensional stability does not equal optimal mechanical performance, as agglomeration defects became mechanical weak points. The 3% AKD sample had the lowest D (1.64 × 10^−6^ mm^2^·s^−1^), indicating that appropriate AKD optimized the interface and maximized diffusion resistance. The 4% AKD sample had the lowest n (0.4786), approaching Fickian diffusion, with agglomeration pores providing fast channels.

These results indicate that interfacial bonding strength is the key factor determining mechanical retention, rather than simply low water absorption or low swelling. When “optimal” is defined as the best balance between mechanical retention and dimensional stability, 2% AKD is optimal for mechanical retention, while 5% AKD is optimal for dimensional stability but requires a trade-off with mechanical degradation. In practical applications, the formulation can be selected within the range of 2–5% according to humid service requirements.

## 4. Conclusions

In this study, poplar wood flour was surface-modified with AKD, and poplar wood flour/PLA composites with different AKD contents were prepared. The effects of AKD content on the wettability, water absorption kinetics, dimensional stability, flexural properties after water absorption, and microscopic interface of the composites were systematically investigated. The main conclusions are as follows:

(1) AKD likely consumed the polar hydroxyl groups on the poplar wood flour surface through esterification and/or hydrogen bonding, as reported in the literature, and introduced hydrophobic long-chain alkyl groups, which increased the contact angle and decreased the total surface free energy of the composites. The 5% AKD sample exhibited the highest contact angle, while the 3% AKD sample showed the lowest total surface free energy (24.73 mJ·m^−2^). The total surface free energy of the 5% AKD sample (24.95 mJ·m^−2^) was slightly higher than that of the 3% AKD sample.
(2) AKD content significantly changed the water diffusion mode. The 0–3% AKD samples exhibited non-Fickian anomalous diffusion, in which water transport was jointly controlled by concentration gradient and wood flour swelling. Among them, the 2% AKD sample had the highest n value (0.713), indicating that swelling-controlled diffusion dominated the water absorption process and the apparent saturation water absorption was the lowest. The 4% AKD sample approached Fickian diffusion, and the agglomerated pores accelerated water transport. The initial apparent diffusion coefficient D was the lowest for the 3% AKD sample, while the 2% AKD sample had a relatively high initial D value; however, later swelling blocked the pores, and its apparent saturation water absorption remained the lowest.
(3) The thickness swelling coefficient first increased and then decreased with increasing AKD content. The 5% AKD sample had the lowest β∞ (10.03%), indicating the best dimensional stability. However, the flexural strength retention was not simply negatively correlated with β∞: the 2% AKD sample did not have the lowest β∞, but exhibited the highest flexural strength retention due to optimal interfacial bonding and the least debonding damage; the 5% AKD sample had the lowest β∞, but agglomeration defects weakened the mechanical properties. Interfacial bonding strength was the key factor determining mechanical retention.
(4) SEM results showed that AKD modification significantly reduced the interfacial gaps between wood flour and PLA matrix and inhibited fiber pull-out. After water absorption, the 2% AKD sample still maintained good interfacial bonding. Using the balance between mechanical retention and dimensional stability as the criterion for “optimal”, 2% AKD is optimal when mechanical retention is prioritized, while 5% AKD is optimal when dimensional stability is prioritized. These findings provide experimental support for the formulation design of PLA-based wood–plastic composites containing bio-based components under humid service conditions.

## Figures and Tables

**Table 1:** Surface tension parameters of the test liquids [16].

Liquid	Surface Free Energy (mJ·m^−2^)	Dispersive Component (mJ·m^−2^)	Polar Component (mJ·m^−2^)
Formamide	58.0	39.5	18.5
Glycerol	64.0	34.0	30.0
Distilled water	72.8	21.8	51.0

**Table 2:** Surface free energy of AKD-modified poplar wood flour/PLA composites.

AKD Content (%)	*γ_S_* (mJ·m^−2^)	$\gamma_{S}^{d}$ (mJ·m^−2^)	$\gamma_{S}^{p}$ (mJ·m^−2^)
0	53.20	6.06	47.14
1	38.72	20.42	18.30
2	27.30	12.83	14.47
3	24.73	8.90	15.83
4	24.93	12.96	11.97
5	24.95	2.42	22.53

**Table 3:** Water absorption kinetics parameters of poplar wood flour/PLA composites.

AKD Content (%)	n	lgK	R^2^	Diffusion Mode
0	0.656	−1.4046	0.9495	Non-Fickian
1	0.622	−1.3344	0.9499	Non-Fickian
2	0.713	−1.5245	0.9589	Non-Fickian
3	0.675	−1.4270	0.9221	Non-Fickian
4	0.479	−1.0233	0.9009	Near-Fickian
5	0.531	−1.1456	0.9662	Near-Fickian

**Table 4:** Initial apparent diffusion coefficients of composites with different AKD contents.

AKD Content (%)	k(×10^−3^ s^−1^/^2^)	R^2^	*D*(×10^−6^mm^2^·s^−1^)
0	1.52	0.8569	2.49
1	1.63	0.8963	2.62
2	1.64	0.9111	2.76
3	1.22	0.6963	1.64
4	1.38	0.8656	2.07
5	1.44	0.9458	2.32

**Table 5:** Thickness swelling coefficients of poplar wood flour/PLA composites at different immersion times.

Time/h	0	1%	2%	3%	4%	5%
1	0.33	0.30	0.26	0.30	0.30	0.25
4	3.48	4.24	4.44	4.54	4.11	4.03
20	6.14	8.14	7.89	7.27	6.46	5.46
30	8.46	10.62	10.11	10.18	8.99	8.10
55	10.28	10.79	10.70	10.91	9.93	8.99
85	11.11	13.96	13.35	13.18	11.98	10.45
134	11.53	12.93	12.37	12.37	11.30	10.03
216	11.61	12.72	12.37	12.37	11.30	10.03

**Table 6:** Flexural strength retention of composites with different AKD contents after saturated water absorption.

AKD Content (%)	Dry Flexural Strength (MPa)	Wet Flexural Strength After 216 h (MPa)	Retention Rate (%)
0	19.40 ± 1.14	5.40	27.84
1	21.75 ± 0.51	8.20	37.70
2	23.33 ± 1.79	10.80	46.29
3	22.40 ± 1.67	8.70	38.84
4	21.40 ± 1.34	7.30	34.11
5	20.00 ± 1.58	7.00	35.00

Note: Values are means of five specimens. Standard deviations for dry strength are reported where replicate data were available; wet-strength SDs and statistical comparisons are not reported because the original replicate data were not retained.

**Table 7:** Comprehensive comparison of water absorption and mechanical properties of composites with different AKD contents.

AKD Content (%)	n	D/(×10^6^mm^2^·s^−1^)	Water Absorption at 216 h (%)	β∞/%	Flexural Strength Retention/%
0	0.656	2.49	12.77	11.61	27.84
1	0.622	2.62	12.74	12.72	37.70
2	0.713	2.76	12.35	12.37	46.29
3	0.675	1.64	14.20	12.37	38.84
4	0.479	2.07	14.69	11.30	34.11
5	0.531	2.32	14.56	10.03	35.00

## Data Availability

The data presented in this study are available in this article. The raw data supporting the conclusions of this article will be made available by the authors on request.

## Supplementary Materials

The following supporting information can be downloaded at: https://www.mdpi.com/article/10.3390/polym18212763/s1, Figure S1. Residual plots for composites with different AKD contents: (a) 0%, (b) 1%, (c) 2%, (d) 3%, (e) 4%, (f) 5%.

## References

[1] Wang Y., Van Putten R.J., Tietema A., Parsons J.R., Gruter G.J.M. (2024). Polyester biodegradability: Importance and potential for optimisation. Green Chem..

[2] Li H.D., Dong H.J., Zhang C.Y., Geng Y., Liu X., Liu G., Zhong C. (2023). Combining LCA-MFA models to identify China’s plastic value chain environmental impact mitigation pathways. iScience.

[3] Ji W.H., Zhang Y.Y., Han T.J., Yang Y., Wang Y., Chen Y.H., Liu D.T. (2026). Research Progress on Cellulose-Polylactic Acid Molecular Composite Strategies. J. Compos. Mater..

[4] Huang C.C., Liu C.Z., Yang R., Mei C.T. (2023). Research Progress on Fused Deposition Printed Micro-Nano Biomass Filler Reinforced Polylactic Acid-Based Composites. J. Compos. Mater..

[5] Shi H.T., Zhao S.Y., Chen L., Tian Y.Y., Li L.F., Cao Y., Han J.Y., Zhang L.R. (2025). Evaluation of GMA-Modified PLA/Bamboo Composites: Mechanical Strength and Biodegradability. ACS Omega.

[6] Plamadiala I., Croitoru C., Pop M.A., Roata I.C. (2025). Enhancing Polylactic Acid (PLA) Performance: A Review of Additives in Fused Deposition Modelling (FDM) Filaments. Polymers.

[7] Liu J.J., Ou W.X., Zeng J.B., Li Y.D. (2025). Preparation and Performance Study of Polylactic Acid/ Walnut Shell Composites. Biomass Chem. Eng..

[8] Zuñiga T.A., Silva F.C.P., Hernandez H.E., Velazquez N.G., Gamez H.J.F., Padilla M.G., Salazar S.E., Nieves S.I. (2025). Plasma-Activated Water/Ultrasound as a Green Method to Modify Wood Fiber By-Product: Insights of Their Mechanical Performance in Polylactic Acid-Based Biofilms. Biopolymers.

[9] Zhao F.Y., Xiang S.F., Zhao S.J., Fu F.Y., Liu X.D. (2024). Research Progress on Pretreated Straw Fibers for Polymer-Based Composites. J. Zhejiang Sci.-Tech Univ..

[10] Parshina A., Yelnikova A., Safronova E., Kolganova T., Bobreshova O., Yaroslavtsev A. (2023). Potentiometric Sensor Arrays Based on Hybrid PFSA/CNTs Membranes for the Analysis of UV-Degraded Drugs. Polymers.

[11] Karagöz İ., Tamer İ.M., Çavuşoğlu A., Sepetcioglu H. (2024). Investigation of mechanical, thermal, and morphological properties of walnut shell and nano clay reinforced HDPE composites. Mater. Today Commun..

[12] Karagöz İ., Karadeniz Y., Sepetçioğlu H., Daştan V., Çınar E. (2026). Hazelnut shell-talc-carbon black hybrid high-density polyethylene biocomposites: Formulation–property relationships for potential rigid food-packaging applications. Polymers.

[13] Liu X.N., Li Y.P., Wang H.L., Song Z.P., Tan C.P., Li G.D., Yu D.H., Liu W.X. (2023). AKD Emulsions Stabilized by Guar Gel: A Highly Efficient Agent to Improve the Hydrophobicity of Cellulose Paper. Polymers.

[14] Feng T.G., Zhu L.J., Zhang R.Y., Xian Y. (2023). Process and Properties of AKD Modified Poplar Powder/PAL Composite. Plastics.

[15] Sannigrahi P., Ragauskas A.J., Tuskan G.A. (2010). Poplar as a feedstock for biofuels: A review of compositional characteristics. Biofuels Bioprod. Biorefin..

[16] Owens D.K., Wendt R.C. (1969). Estimation of the Surface Free Energy of Polymers. J. Appl. Polym. Sci..

[17] Lin W.X., Li X.Y., Li L., Li S.J. (2025). Preparation and performance study of cellulose nanofiber/polylactic acid composite films. J. Cellul. Sci. Technol..

[18] Wan H., Sun C., Xu C., Wang B.W., Chen Y., Yang Y.Q., Zhang Y.H. (2024). Synergistic reinforcement of polylactic acid/wood fiber composites by cellulase and reactive extrusion. J. Clean. Prod..

[19] Yu L.X., Qiu Y., Yang B., Gao M., Song L.X., Jing Y., Chi W.H., Wang X.Y., Zhang L.W., Gao J.L. (2024). Developing high-performance and sustainable polylactic acid/recycled polyolefin blends: Tuning the degree of functional group reaction and performance optimization. Int. J. Biol. Macromol..

[20] Asad F., Immonen K., Kiiskinen T., Mikkelson A., Sarlin E. (2025). The Impact of Mechanical Recycling on Ligno-Cellulose Fibre Containing PLA Biocomposite. Polymers.

[21] Akilah F.A., Mokhtar S.M. (2024). Tailoring alkyl ketene dimer on structural-properties relationship of cellulose-based materials: A short review. Polym.-Plast. Technol. Mater..

[22] Shen R.S., Wang D.F., Chen Y.L., Liu L. (2023). Green Separation of Wood Fibers and the Performance Study of Their Reconstituted Composites. Biomass Chem. Eng..

[23] Tang Z.W., Wang S.S., Kong D.Z., Zhang Y., Liu S., Li L.H., Li R. (2024). Effects of Fillers and Additives on AKD Sizing. J. Northeast Agric. Univ..

[24] Jerič P., Golja B., Lavrič G., Teržan J., Verbič A., Likozar B., Novak U. (2026). Sustainable, Safe, and Effective (Super)Hydrophobic Coatings for Cellulosic Fiber Material via Alkyl Ketene Dimer and Polysaccharide Integration. ACS Sustain. Chem. Eng..

[25] Wikoramet T., Khemthat S., Pattara S. (2024). Development of Biodegradable Poly(Lactic Acid)/Lignin Treated Alkyl Ketene Dimer Properties for Packaging Applications. Adv. Sci. Technol. Res. J..

[26] Hong Q.Y., Wang H.Y., Li Y., Li R.Q., Deng Q.Y., Liu C.X., Dai H.Q. (2025). Study on the Preparation of New Wood-Plastic Composites Using Micro-Nano Fiber Modified Polyethylene Pellets. J. Compos. Mater..

[27] Zhang R.Y., Peng H.X., Li S.H., Yang X., Li H.B., Xing Z.B., Xian Y. (2025). Effect of biochar content and particle size on mechanical and water absorption properties of landscaping waste/polylactic acid composites. Ind. Crops Prod..

[28] Ju Z.H., Wang Z.Q., Zhang H.Y., Zheng W., Shu B.Q. (2024). Thermal and Mechanical Properties of 3D-Printed Polyethylene Glycol-Modified Lignin/Polylactic Acid Biocomposites. J. Compos. Mater..

[29] Ren T.X., Xu Y.D., Zhan Y.T., Zhan H.H., Shao Y.Y., Wu Z.P., Zhang K., Chen L.F., Shen W., Ouyang Y.W. (2025). Interfacial and Optical Properties of Plasma-Modified Optically Transparent Composites With Sandwich-Structure Based on Waste Textiles. Polym. Compos..

[30] Song H.L., Hon C.K., Gauvin F., Pantaleo S., Berger F., Chen W., Brouwers H.J.H. (2025). Durability assessment of alkyl ketene dimer hydrophobic treatment of bio-based thermal insulation materials. Resour. Conserv. Recycl..

[31] Wang B., Liang Y.H., Wang X. (2024). Preparation and Performance of Salix/Wheat Straw Composite Fiber Mulch. China For. Prod. Ind..

[32] Xu W.L., Li M.Y., Xu Y.N., Entezari A., Fang J.G. (2025). Mechanical Characterization of Fused Deposition Modeling-Printed Wood-Polylactic Acid Composites Under Water Conditioning. Polym. Compos..

[33] Zhang X.M., Wang X., Hu J.P. (2023). Research Progress on the Aging Mechanism of Wood-Plastic Composites and Improvement of Aging Resistance. World For. Res..

[34] Wang Y.T., Huang Y.F., Zhong J., Yu C.H. (2023). Superhydrophobic Coatings on Cellulose-Based Materials with Alkyl Ketene Dimer Pickering Emulsion: Fabrication and Properties. Coatings.

