# Supplementary material for: Effect of AKD Content on Wettability, Water Absorption Kinetics, and Interfacial Properties of Poplar Wood Flour/PLA Biocomposites
Source: Polymers (Basel). 2026 Nov 6;18(21):2763. doi: 10.3390/polym18212763 (PMC13765700; doi:10.3390/polym18212763)
Supplement: Supplementary file 1 [file polymers-18-02763-s001.zip › polymers-4659615-supplementary.pdf]

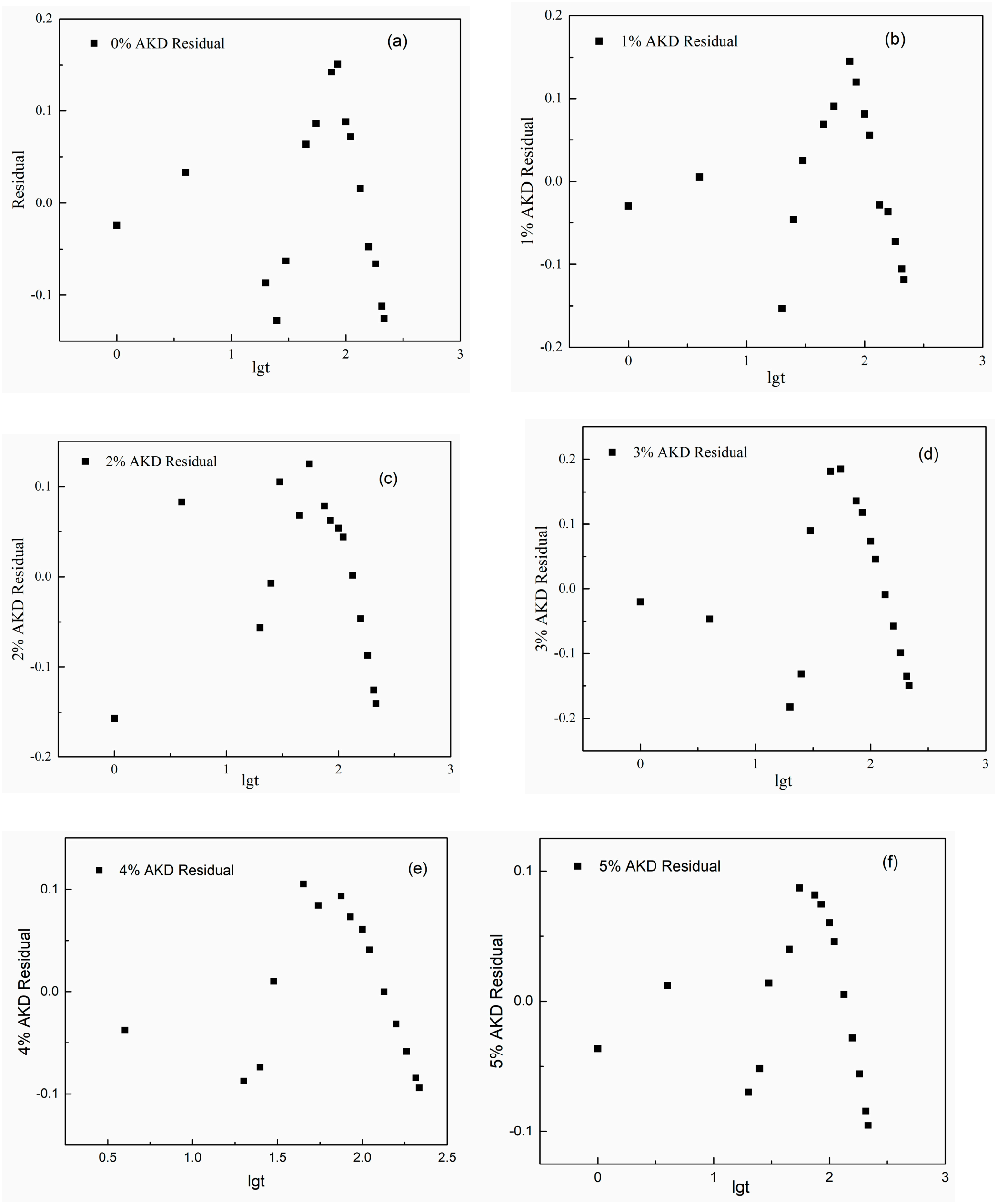

Figure S1. Residual plots for composites with different AKD contents: (a) 0%, (b) 1%, (c) 2%, (d) 3%, (e) 4%, (f) 5%.
